# An Adult Developmental Approach to Perceived Facial Attractiveness and Distinctiveness

**DOI:** 10.3389/fpsyg.2018.00561

**Published:** 2018-05-07

**Authors:** Natalie C. Ebner, Joerg Luedicke, Manuel C. Voelkle, Michaela Riediger, Tian Lin, Ulman Lindenberger

**Affiliations:** ^1^Department of Psychology, University of Florida, Gainesville, FL, United States; ^2^Department of Aging and Geriatric Research, Institute on Aging, University of Florida, Gainesville, FL, United States; ^3^Department of Clinical and Health Psychology, Center for Cognitive Aging and Memory, University of Florida, Gainesville, FL, United States; ^4^Center for Lifespan Psychology, Max Planck Institute for Human Development, Berlin, Germany; ^5^Department of Psychology, Humboldt University of Berlin, Berlin, Germany; ^6^Department of Psychology, University of Jena, Jena, Germany; ^7^Department of Political and Social Sciences, European University Institute, Fiesole, Italy

**Keywords:** age, emotion, faces, attractiveness, distinctiveness, cross-classified random effects analysis

## Abstract

Attractiveness and distinctiveness constitute facial features with high biological and social relevance. Bringing a developmental perspective to research on social-cognitive face perception, we used a large set of faces taken from the FACES Lifespan Database to examine effects of face and perceiver characteristics on subjective evaluations of attractiveness and distinctiveness in young (20–31 years), middle-aged (44–55 years), and older (70–81 years) men and women. We report novel findings supporting variations by face and perceiver age, in interaction with gender and emotion: although older and middle-aged compared to young perceivers generally rated faces of all ages as more attractive, young perceivers gave relatively higher attractiveness ratings to young compared to middle-aged and older faces. Controlling for variations in attractiveness, older compared to young faces were viewed as more distinctive by young and middle-aged perceivers. Age affected attractiveness more negatively for female than male faces. Furthermore, happy faces were rated as most attractive, while disgusted faces were rated as least attractive, particularly so by middle-aged and older perceivers and for young and female faces. Perceivers largely agreed on distinctiveness ratings for neutral and happy emotions, but older and middle-aged compared to young perceivers rated faces displaying negative emotions as more distinctive. These findings underscore the importance of a lifespan perspective on perception of facial characteristics and suggest possible effects of age on goal-directed perception, social motivation, and in-group bias. This publication makes available picture-specific normative data for experimental stimulus selection.

## Introduction

Facial attractiveness constitutes salient facial features with high biological and social relevance. Perceptions of both facial attractiveness and distinctiveness impact thought and behavior in various contexts (Eagly et al., 1991; Penton-Voak et al., 1999; Wickham and Morris, 2003; Griffin and Langlois, 2006; Wiese et al., 2014; Lin et al., 2016).

Facial attractiveness serves as biological marker of reproductive fitness, influencing mating success and kinship opportunities (Penton-Voak et al., 1999; Thornhill and Gangestad, 1999; Havlicek et al., 2005; Feinberg et al., 2006; Puts et al., 2013). It is processed automatically and characterized by a high degree of inter-individual and inter-cultural consistency (Perrett et al., 1994; Slater et al., 1998; Langlois et al., 2000). Facial attractiveness also constitutes a social construct, with attractive people experiencing advantages. For example, in line with the beauty-is-good-effect, attractive people are perceived as more positive and likeable and receive increased altruism (Dion et al., 1972; Eagly et al., 1991; Griffin and Langlois, 2006). Also, performance of attractive people is typically evaluated more favorarably, with consequences for academic success and employment prospects, job promotion, and salary paid (Landy and Sigall, 1974; Benson et al., 1976; Agthe et al., 2011). These effects may be due to the strong affective response attractiveness elicits in the perceiver, as supported by evidence that the fundamental biological reward circuitry is involved in processing attractiveness (Nakamura et al., 1998; Aharon et al., 2001; Kampe et al., 2001; O’Doherty et al., 2003). This reward processing associated with attractive faces may also underlie improved memory for more attractive faces (Marzi and Viggiano, 2010; Zhang et al., 2011; Lin et al., 2016). Further, structural facial features such as symmetry and facial distinctiveness, the extent to which a face deviates from the typical face (i.e., distance from the norm), have been shown to affect perceived attractiveness (Grammer and Thornhill, 1994; Rhodes and Tremewan, 1996; Rhodes et al., 1999; Hume and Montgomerie, 2001; Wickham and Morris, 2003; Valentine et al., 2004; Zaidel and Cohen, 2005; Komori et al., 2009).

### Effects of Face and Perceiver Characteristics on Perception of Facial Attractiveness and Distinctiveness

Growing evidence suggests that features of the face (e.g., age, gender, emotion; Alicke et al., 1986; Furnham et al., 2001; Ebner, 2008; Foos and Clark, 2011; Kwart et al., 2012) and perceiver characteristics (e.g., age, gender; Ebner, 2008; Foos and Clark, 2011) influence perception of both facial attractiveness and distinctiveness, with subsequent cognitive and behavioral effects (attention, memory performance, personality evaluation, mate selection, etc.). A thorough integration of lifespan developmental and social-cognitive research on this topic, however, is currently lacking. To fill this gap, this study determined the extent to which face (age, gender, emotion) and perceiver (age, gender) characteristics affect subjective perception of facial attractiveness and distinctiveness. In the following, we summarize the current knowledge regarding effects of these face and perceiver characteristics on attractiveness and distinctiveness ratings. Each section concludes with a presentation of our specific study hypotheses based on theory and previous empirical work.

### Age of Face and Perceiver Effects

Age is a relevant factor in face processing (Shepherd et al., 1981; George and Hole, 1998; Benson, 1995). Youthfulness of faces has been associated with increased attractiveness (Mathes et al., 1985; Alley, 1988; Henss, 1991; Zebrowitz et al., 1993). That is, typically, older adult faces are perceived as less attractive than young adult faces (Wernick and Manaster, 1984; Kissler and Bäuml, 2000; Furnham et al., 2004; Ebner, 2008; Foos and Clark, 2011; Kwart et al., 2012). Relatedly, unattractive faces are rated as older than age-matched attractive faces (Kwart et al., 2012). These effects may be due to changes in shape, which mainly occur through growth or weight gain or loss, and changes in the characteristics of the surface texture and coloration of skin and hair associated with aging (Burt and Perrett, 1995; Bruce and Young, 1998; Ebner, 2008).

In contrast, effects of age of face on facial distinctiveness are largely unexplored. One previous study suggests that older but not young perceivers rated older faces as less distinctive than young faces, in line with an in-group familiarity account (Ebner, 2008; see also Bartlett and Fulton, 1991). In-group favoritism (Mullen et al., 1992), the mere-exposure effect (Mita et al., 1977), or prototyping (Corneille et al., 2007) could explain why faces from the own-age group may be perceived as more familiar and thus more average and hence less distinctive than faces from other age groups. However, increased facial caricature distortion can also result in perception of increased age of the face (O’Toole et al., 1997), possibly because certain facial features become more distinctive with age. This latter evidence suggests that older faces should be perceived as more distinctive than young faces.

In addition, perceiver characteristics, needs, and goals (Gibson, 1979; McArthur and Baron, 1983), social stereotypes (Hummert et al., 1994; Gluth et al., 2010), and cognitive-perceptual abilities affect face perception. In particular, age-related change in primary social motivation and age differences in the frequency of everyday exposure to faces of different ages may influence the relevance of facial attractiveness and distinctiveness in adults of different ages. In particular, Socioemotional Selectivity Theory (Carstensen et al., 1999; Mather and Knight, 2005; Isaacowitz et al., 2006) proposes that content and prioritization of social goals change across adulthood. Older adults typically report higher frequency and more positive experiences in their social interactions with close others (e.g., family members; Carstensen, 2006). In contrast, young adults typically report higher frequency of and more positive experiences in social interactions with new friends (Charles and Piazza, 2007). This is in line with the idea that age-graded developmental tasks change across the adult lifespan (Heckhausen et al., 2010), with age-relevant goals toward family building typically holding priority in young adulthood (Nurmi, 1992; Hooker and Kaus, 1994; Frazier et al., 2000). Considering this primary social motivation in young adulthood of forming new friendships (Fredrickson and Carstensen, 1990) and developing romantic partnerships (Erikson, 1966; Zimmer-Gembeck, 2002), we propose that face attractiveness constitutes a particularly relevant feature in young adults’ subjective face perception. In contrast, facial attractiveness may play less of a role in older adults’ processing of particularly unfamiliar faces, as their primary social focus is on close relationships with familiar others.

Typically, older perceivers’ rate faces as more attractive than young perceivers (Ebner, 2008). However, controlling for this main effect, there is evidence of a self-serving bias, that is, a tendency of individuals to attribute positive features to their own age group and distance from negative characteristics in a self-promoting manner (Mezulis et al., 2004; Heider, 2013; Lin et al., 2017). Accordingly, lower attractiveness ratings for older compared to young faces appear relatively less pronounced in older than young perceivers (Wernick and Manaster, 1984; McLellan and McKelvie, 1993; Ebner, 2008; Foos and Clark, 2011).

Based on these considerations, for facial attractiveness we predicted that older compared to middle-aged and particularly young perceivers rated faces as more attractive (*Hypothesis 1a*; *main effect*: *age of perceiver*). We expected higher attractiveness ratings for young compared to middle-aged and particularly older faces (*Hypothesis 1b*; *main effect*: *age of face*). We expected this effect to be most pronounced in young perceivers (*Hypothesis 1c*; *interaction effect*: *age of face* × *age of perceiver*).

Regarding age effects on subjective perception of facial distinctiveness, it is relevant to consider that young adults typically report great frequency of everyday contact with young persons, while they have less frequent everyday exposure to older persons (Ebner and Johnson, 2009). In contrast, older adults typically report more comparable frequency of contact with young and older persons. This may be due to greater representation of young compared to older faces in the media (e.g., on TV, in magazines). Thus, it is plausible to assume that, given their comparable familiarity with own-age and other-age faces, older adults do not, or at least less than young adults, differentiate between levels of distinctiveness based on the age of a face.

Based on these considerations, for facial distinctiveness, we predicted that older compared to middle-aged and particularly young faces were perceived as more distinctive (*Hypothesis 2a*; *main effect*: *age of face*). We expected this effect to be more pronounced in young compared to middle-aged and particularly older perceivers (*Hypothesis 2b*; *interaction effect*: *age of face* × *age of perceiver*).

Both attractiveness and distinctiveness can be determined objectively (e.g., via facialmetric assessment), with downstream effects on cognition and behavior. However, attractiveness and distinctiveness also constitute subjective evaluation criteria (e.g., based on personal taste and experience and familiarity) associated with cognitive and behavioral effects (e.g., on memory, in social interactions). The majority of current research has aimed at obtaining objective measures of attractiveness and distinctiveness, such as related to facial symmetry or mathematical deviation of an individual face from the average face (Bookstein, 1991; Rhodes et al., 1999; Komori et al., 2009). To guard against possible bleed-through effects from perception of attractiveness on perception of distinctiveness, especially when assessed subjectively and in close sequence of each other as in the present study, we controlled for variations in attractiveness in all analyses pertaining to distinctiveness. This allowed us to dissociate effects on attractiveness from those on distinctiveness.

### Gender of Face and Perceiver Effects

Gender is another relevant factor in perceived facial attractiveness and distinctiveness (Bruce and Young, 1986; Baudouin and Gallay, 2006). There are particular sexually dimorphic features such as thick brow ridges and a large jaw structure in male faces, and small lower face, high cheekbones, and thick lips in female faces that are associated with gender-specific attractiveness (Perrett et al., 1998). These facial features have been shown to serve as hormone markers and markers of genetic health and fertility, and hence reproductive fitness (Penton-Voak et al., 1999; Thornhill and Gangestad, 1999; Havlicek et al., 2005; Feinberg et al., 2006; Puts et al., 2013).

Effects of social categorization and prototyping of faces (Valentine and Ferrara, 1991) further suggest that gender of face may play a role in face perception. For example, average features of faces have been shown to contribute to increasing a face’s perceived attractiveness, but only when these features are average within the group to which the face belongs (Potter and Corneille, 2008). There also is supportive evidence that subjective distinctiveness ratings are based on comparisons with the population of same-gender faces rather than on comparisons with an overall reference across genders (Baudouin and Gallay, 2006).

Supporting the importance of considering gender of perceiver effects, inter-rater agreement regarding the attractiveness of sexually dimorphic features is especially high when men rate female faces (Marcus and Miller, 2003). Furthermore, gender of face by gender of perceiver interactions are supported by gender-differential neural recruitment patterns during face viewing. Medial prefrontal cortex (O’Doherty et al., 2003) and nucleus accumbens and orbitofrontal cortex (Aharon et al., 2001), regions associated with affective and reward processing (Delgado et al., 2000; Rolls, 2000), were particularly activated when men viewed attractive female faces. Accordingly, Aharon et al. (2001) showed that while men rated both male and female faces as attractive (i.e., aesthetic value; “liking”), they were only willing to exert effort (measured as the willingness to continue pressing keys) to prolong views of attractive female but not male faces (i.e., reward value; “wanting”).

Of note, the few studies that have examined interaction effects of gender of face and perceiver with age of face and perceiver found that while both young and older raters perceived young female faces as more attractive than male faces (Wernick and Manaster, 1984), older women were rated least attractive, particularly by young perceivers (McLellan and McKelvie, 1993; Foos and Clark, 2011). The present study aimed to further explore these developmentally relevant but currently still understudied age-by-gender interaction effects.

Considering this previous research, for facial attractiveness, we expected that particularly for female faces, young faces were rated as more attractive than middle-aged and older faces (see *Hypothesis 1b*) (*Hypothesis 1d*; *interaction effect*: *age of face* × *gender of face*). Given the limited literature on gender effects on facial distinctiveness, we did not formulate specific hypotheses pertaining to this rating dimension.

### Emotion of Face Effects

Facial expressions are used to display mood (Tompkins, 1962; Izard, 1971; Darwin et al., 1998). There is evidence for a modulatory effect of facial emotion on ratings of attractiveness. In particular, smiling faces were evaluated as more attractive than neutral faces (Otta et al., 1996) and individuals appeared less attractive when their facial expression was sad compared to neutral or happy (Mueser et al., 1984). This may be because happy expressions generate a positive affective response in the perceiver, who might then see the other person as more attractive. Accordingly, increased activity of the orbitofrontal cortex in response to high attractive compared to low attractive faces was particularly pronounced for smiling faces (O’Doherty et al., 2003). In contrast, activity in the insula, and other brain regions previously associated with processing of negative facial emotion (e.g., disgust; Ruiz et al., 2013), was greater in response to low attractive than high attractive faces.

In a given face, smiling constitutes one of the most important factors in determining attractiveness and this positive association holds in young, middle-aged, and older raters (Foos and Clark, 2011). Importantly, however, accuracy in emotion recognition varies as a function of the age of the face and the perceiver, supporting the notion that effects of emotion on facial attractiveness need to be addressed from a developmental perspective. In particular, emotions were generally harder to read in older compared to young faces (Ebner and Johnson, 2009; He et al., 2011; Riediger et al., 2011). In addition, both young and older perceivers were more likely to attribute emotional compared to neutral expressions to older relative to young faces (Ebner, 2008). This may be because of greater ambiguity of emotional display in older faces caused by facial wrinkling, decrease in muscle flexibility, or change in social display rules (Fölster et al., 2014). Also, older compared to young adults have more difficulty with emotion recognition (Ruffman et al., 2008; Ebner et al., 2010) and older adults show a bias in that they are more likely than young adults to attribute unintended positive and neutral expressions to faces while they are less likely to attribute intended negative expressions (Riediger et al., 2011). Thus, based on evidence that smiling faces appear more attractive (Reis et al., 1990), and that attractiveness is closely related to youth (Henss, 1991), in the present study, we considered facial emotion in affecting perception of facial attractiveness.

Based on previous evidence, for facial attractiveness, we hypothesized that happy faces were rated as most attractive (*Hypothesis 1e*; *main effect*: *emotion of face*), with this effect particularly pronounced in young compared to middle-aged and older faces (*Hypothesis 1f*; *interaction effect age of face* × *emotion of face*).

To our knowledge, no current research exists on effects of emotion of face on perceived distinctiveness. The closest evidence of a relationship between emotion of face and facial distinctiveness comes from Calvo and Marrero (2009) who showed that distinctiveness of local facial features such as showing of teeth promoted face pop-out effects. Given that expressions of happiness are often accompanied by showing teeth, happy faces may therefore be perceived as more distinctive than faces displaying other emotion expressions. However, while pop-out effects represent an automatic detection capacity, perception of facial distinctiveness as examined in the present study is based on a subjective conscious judgment. Thus, for facial distinctiveness, we hypothesized greater distinctiveness ratings for positive compared to negative facial emotions (*Hypothesis 2c*; *main effect*: *emotion of face*).

The current literature allows formulation of hypotheses pertaining to the interactions between age and emotion on facial attractiveness and distinctiveness. However, even though a discrete emotions perspective to emotional aging (Kunzmann et al., 2014) proposes distinct age differences for anger and sadness, currently the empirical and theoretical basis is not sufficient to propose age-differential hypotheses pertaining to effects of specific negative emotions on perceptions of facial attractiveness or distinctiveness.

### The Present Study

The present study extended previous research by acquiring ratings of both attractiveness and distinctiveness for a large set of young, middle-aged, and older male and female naturalistic face stimuli from a large sample of young, middle-aged, and older women and men to pursue the following three aims:

(1) Examination of face and perceiver effects on perception of attractiveness and distinctiveness. With this article we followed up on published work with the same dataset in which we examined age-of-perceiver and age-of-face effects on emotion perception (Ebner et al., 2010; Riediger et al., 2011) and age estimation (Voelkle et al., 2012). Using unpublished information, the present study goes beyond this previous work by examining face and perceiver effects on attractiveness and distinctiveness ratings, a link that we had not established before.

(2) Exploration of relations between facial attractiveness and distinctiveness. The current literature has addressed the attractiveness-distinctiveness relationship from different theoretical angles, generating mixed evidence. For example, evolutionary psychology argues that people with average (i.e., less distinctive) facial features are seen as attractive, because the *averageness* may reflect absence of genetic mutation and thus may be indicative of greater health and thus greater potential for healthy offspring (Thornhill and Gangestad, 1999). This is based on reasoning that average people have the best chance of survival and thus one’s own fitness is maximized by mating with an average partner. This results in selection pressure for the attractiveness of averageness. Similarly, suggesting a linear relationship between facial attractiveness and distinctiveness, the “prototypicality” account of attraction argues that objective prototypicality drives increased perception of attractiveness due to ease of processing (Light et al., 1981; Mueller et al., 1984; Langlois and Roggman, 1990; Rhodes and Tremewan, 1996).

However, evidence that attractive, but not very attractive, faces were perceived as average speaks against such linear relationship between attractiveness and distinctiveness (Alley and Cunningham, 1991). In addition, certain objective distinctive facial features, as determined via facialmetric assessments (e.g., larger than average eyes, smaller than average nose and chin for women, and larger than average eyes, cheekbones, and chins for men), were perceived as particularly attractive (Cunningham, 1986; Cunningham et al., 1990), likely in their function of reflecting hormone status and reproductive fitness (Penton-Voak et al., 1999; Thornhill and Gangestad, 1999; Puts et al., 2013). This evidence entertains the idea that certain face and perceiver characteristics need to be taken into consideration when evaluating perception of facial attractiveness and distinctiveness. Thus, an exploratory aim of the present study was to inform the currently mixed evidence on the relation between attractiveness and distinctiveness ratings, and to consider variations by age and emotion in this context.

(3) Acquisition of picture-specific norm data. Ebner et al. (2010) described the development and validation of the FACES Lifespan Database. As part of the normative data collected for the FACES database validation, the dimensions of facial attractiveness and distinctiveness were assessed but had not been published in Ebner et al. (2010). Therefore, the present study also had the practical goal to make available raw means and standard deviations of attractiveness and distinctiveness ratings for each face to permit researchers the selection of experimental stimuli from the FACES database based on picture-specific normative information.

## Materials and Methods

### Participants

Through the institute’s participant pool and word-of-mouth, 154 White, German-speaking adults were recruited into the study, of which 52 were young (*M* = 26.0 years, *SD* = 2.95, 20–31 years, 48% female), 51 middle-aged (*M* = 50.0 years, *SD* = 3.40, 44–55 years, 51% female), and 51 older (*M* = 73.6 years, *SD* = 2.75, 70–81 years, 53% female). Details about the demographic composition of the final sample are given in Ebner et al. (2010).

The age by gender groups of participants did not differ in their self-reported physical functioning (single item: *How would you describe your current physical functioning*?; scale of 1–8, with 8 = *excellent*; *M* = 5.5, *SD* = 1.5) but differed in their visual-motor processing speed as assessed by the Digit Symbol Substitution test (Wechsler, 1981): Young women (*M* = 66.3, *SD* = 11.1) and young men (*M* = 64.0, *SD* = 9.6) scored higher than middle-aged women (*M* = 46.0, *SD* = 9.1) and middle-aged men (*M* = 48.5, *SD* = 14.4) and higher than older women (*M* = 44.8, *SD* = 10.7) and older men [*M* = 47.7, *SD* = 12.1; *F*(5,143) = 18.3, *p* < 0.05, $\eta_{p}^{2}$ = 0.39; max score = 93]. Individuals in the middle-aged and older groups did not significantly differ in their visual-motor processing speed. This pattern is in line with typical performance differences in these age groups.

### Stimuli

Face stimuli comprised photographs of 58 young (19–31 years), 56 middle-aged (39–55 years), and 57 older (69–80 years) individuals, taken from the FACES Lifespan Database (Ebner et al., 2010). FACES is a unique database, comprised of a large (*N* = 171) set of naturalistic faces of young, middle-aged, and older women and men. Images are in color with hairstyles ears, and shoulders included, as opposed to “egg faces” (with cropped hair, ears, and other surrounding features). Each face identity in the database is represented in two parallel sets (Set A and Set B) of six prototypical facial expressions (neutrality, sadness, disgust, fear, anger, happiness; in line with Ekman and Friesen, 1975; Ekman, 1993, excluding surprise because surprise and fear are frequently confounded). This resulted in 2,052 individual high-quality color photographs. The two-set database feature allows researchers to use face images of the same identity and facial expression that are not the same images, which may be a requirement for certain study designs (e.g., in memory paradigms).

### Procedure

**FIGURE 1:**
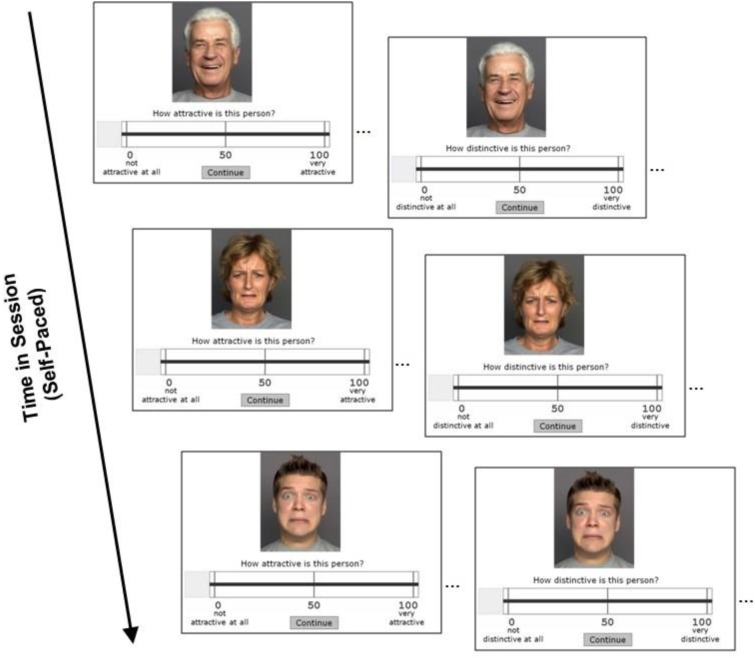
Illustration of the rating procedure. During each session, participants rated the attractiveness and the distinctiveness of different face pictures by adjusting a slider on a scale ranging from 0 to 100. Written informed consent has been obtained from depicted individuals for the publication of the images.

Data collection for the current data analysis was conducted in the context of a larger project. The ethics review board of the Max Planck Institute for Human Development approved the study. All participants gave consent for study enrollment. Participants were informed that in the face rating task they were presented with faces of different people and were asked to view each face carefully and evaluate it on a series of rating dimensions on scales from 1 to 100. Participants were explicitly told that we were interested in their personal opinion, that there were no right or wrong answers, and were asked to give a spontaneous response. Participants completed one face at a time, presented in randomized order, before moving on to the next face in a self-paced fashion. Rating dimensions were presented in the following order: Attractiveness (*How attractive is this person*?), age (*How old is this person*?), distinctiveness (*How distinctive is this person*?), growth orientation (*How much would this person like to improve abilities*?), loss-prevention orientation (*How much would this person like to prevent losses in abilities*?), and facial expression (*neutral, happy, sad, angry, fearful, or disgusted*, order randomly mixed). In the present study, we were interested in the attractiveness and distinctiveness ratings, as shown in **Figure 1**. If participants further inquired what attractiveness referred to, they were told that it referred to “handsome-looking”; terms like “likeability” or “sexually attractive” were not mentioned. No additional information was provided for distinctiveness as participants did not further inquire about it. Before starting the main task, participants worked on practice trials for task familiarization.

As noted, one goal of this project was to provide picture-specific norm data of attractiveness and distinctiveness ratings for each face of the original FACES database to facilitate controlled stimulus selection. Thus, we obtained respective values for each physical image (from both sets) for the norm data report. However, we randomly assigned participants to one of the two sets of parallel face pictures to counter fatigue effects (we had not theoretical or empirical reason to assume responses to images from the two different sets to vary). Face pictures were presented one at a time at the size of 19 × 16 cm, displayed on a 19-inch monitor (screen size, 1,280 × 1,024 pixels). Thus, each participant was asked to rate up to a maximum of 1,026 images.

To further counter fatigue effects, participants did not complete all ratings in a single session, but rather across several sessions spanning over various days. Each session was terminated after 100 min, and there was only one session per day. To further maintain concentration throughout the task, the program stopped for 5-min breaks after every 45 min. A maximum of *M* = 88.03 images (*SD* = 29.99; range: 1–206) were rated per session. On average, it took participants 11.3 (*SD* = 4.7) sessions (days) to complete all ratings. There were individual differences, but the majority of participants completed all ratings within 5–15 days. After the first three sessions, participants were free to discontinue their participation at any point, without forfeiting financial reimbursement. However, only a very small number of participants opted for this: 11 people (7%; 7 middle-aged and 4 older participants) provided less than 25% of the maximum number of ratings. Given the variation in number of pictures rated and sessions across participants, we accounted for within-subject variability across sessions by including a corresponding random effect, and we controlled for number of pictures rated per session in our models. Thus, other model parameters can be interpreted as if every participant rated the same number of pictures and participated in the same number of sessions.

Stimulus presentation was controlled using custom-made software on Pentium (R) 4 CPU 2.8 GHz computers. Participants responded to a short socio-demographic questionnaire including one item on physical functioning and worked on the Digit-Symbol-Substitution test. At the end of the study, participants were debriefed and received a monetary compensation that varied according to the length of their participation in the study. In particular, the amount depended on the number of face pictures rated and on the number of testing sessions attended, and ranged between 50 and 342 EUR.

### Data Analysis

We used multilevel random intercept models (Gelman and Hill, 2007). Specifically, we used a cross-random effects analysis with cross-classification of perceivers and faces, and a nesting structure for repeated observations within perceivers. By using a crossed random-effects design with additional random effects for repeated ratings, we allowed the ratings made by the same perceivers to be correlated over time, as well as allowed for dependencies of ratings of the same pictures made by different perceivers. In addition, a multilevel approach allowed us to directly model the error variances pertaining to the cross-classification and the hierarchical nature of the dataset, leading to more accurate inferential estimates as opposed to, for example, a conventional single-level regression model. In a conventional regression-modeling framework, we would have had to assume that all ratings from the same person and ratings of the same picture by different persons would be independent with equal error variance. These assumptions would have been unrealistic given the complexity of the present study.

Our basic error-component model (M1) can be denoted as follows:

$$y_{ijt}=\beta_{0}+\zeta_{i}+\zeta_{j}+\zeta_{it}+\varepsilon_{ijt}$$

where *y_ijt_* is the outcome (i.e., attractiveness and distinctiveness ratings, respectively) of the *i*th perceiver for the *j*th picture at the *t*th session, and β_0_ is the constant. ζ*_i_* is the variance component for the *i*th perceiver, ζ*_j_* the error term for the *j*th picture, ζ*_it_* is the error term capturing variation over study sessions within the *i*th perceiver, and ε*_ijt_* is the residual error.

In addition to the error-components model, we fitted, for each of the two outcome variables (attractiveness and distinctiveness, respectively), a model including predictor variables of interest (M2). The model with attractiveness as response variable can be denoted as:

$$y_{ijt}=\beta_{0}+\beta_{1}X_{i}+\beta_{2}X_{j}+\beta_{3}X_{it}+\zeta_{i}+\zeta_{j}+\zeta_{it}+\varepsilon_{ijt}$$

and the model with distinctiveness as response variable can be denoted as:

$$y_{ijt}=\beta_{0}+\beta_{1}X_{i}+\beta_{2}X_{j}+\beta_{3}X_{it}+\beta_{4}X_{ijt}+\zeta_{i}+\zeta_{j}+\zeta_{it}+\varepsilon_{ijt}$$

where *X_i_* is a vector of covariates for the *i*th perceiver (i.e., two dummy variables for age of perceiver and one dummy variable for gender of perceiver), *X_j_* is a vector of covariates for the *j*th picture (i.e., dummy variables for age, gender, and emotion of face), and *X_it_* are rating counts (i.e., the number of faces that were rated in a given session) for the *i*th perceiver at point *t*. Rating counts were modeled as continuous covariates with an additional squared term for rating counts to allow for some flexibility in the fitted regression line (which appeared to be a reasonable choice based on exploratory analyses). Thus, all model estimates were adjusted for the varying number of rated pictures. Further, to control for a possible “halo” effect from attractiveness to distinctiveness ratings, all analyses pertaining to distinctiveness were controlled for variations in attractiveness. The (transposed) vectors of the fixed effects parameters to be estimated are denoted as β.

**FIGURE 2:**
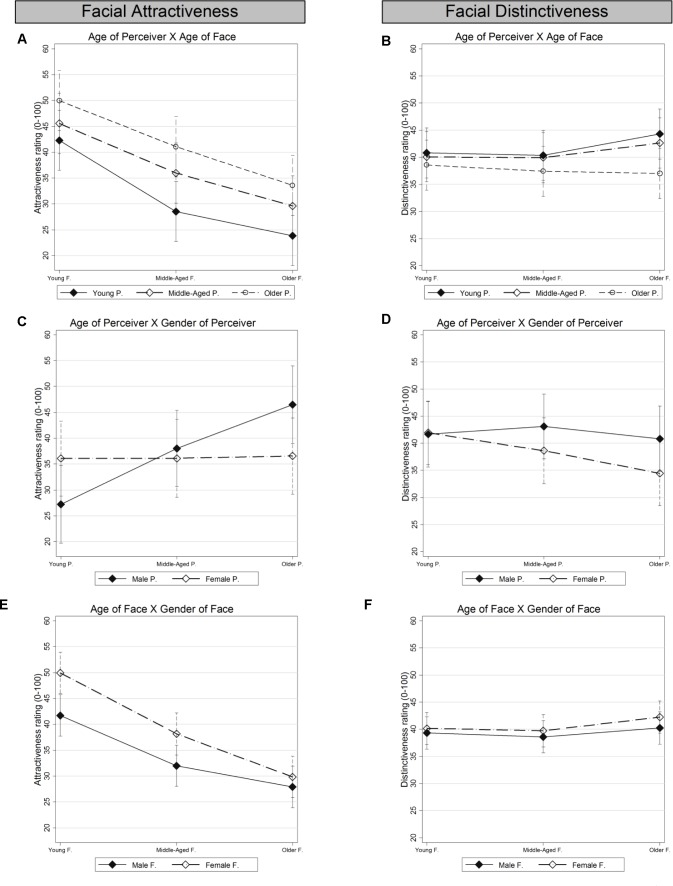
Predicted marginal means for attractiveness and distinctiveness for specified two-way interactions pertaining to age and gender: **(A)** Age of Perceiver × Age of Face, for attractiveness. **(B)** Age of Perceiver × Age of Face, for distinctiveness. **(C)** Age of Perceiver × Gender of Perceiver, for attractiveness. **(D)** Age of Perceiver × Gender of Perceiver, for distinctiveness. **(E)** Age of Face × Gender of Face, for attractiveness. **(F)** Age of Face × Gender of Face, for distinctiveness. Error bars indicate 95% confidence intervals for the point predictions, corrected for multiple comparisons (Bonferroni). P = Perceiver; F = Faces.

**FIGURE 3:**
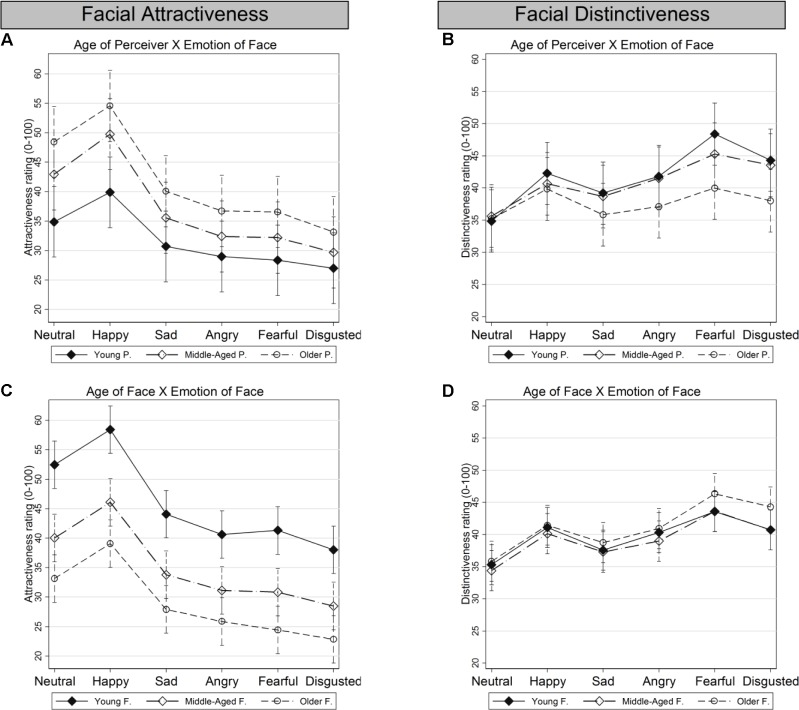
Predicted marginal means for attractiveness and distinctiveness for specified two-way interactions pertaining to age, gender, and emotion of face: **(A)** Age of Perceiver × Emotion of Face, for attractiveness. **(B)** Age of Perceiver × Emotion of Face, for distinctiveness. **(C)** Age of Face × Emotion of Face, for attractiveness. **(D)** Age of Face × Emotion of Face, for distinctiveness. Error bars indicate 95% confidence intervals for the point predictions, corrected for multiple comparisons (Bonferroni). P = Perceiver; F = Faces.

In addition, models for each of the two outcome variables – facial attractiveness and facial distinctiveness – were fitted that included several interaction effects of interest. Parameters of all models were estimated by maximum likelihood. Although both outcome variables were rather uniformly distributed over the bounded range [0,100], the conditional outcome distributions (i.e., the residual errors) appeared to be approximately normally distributed without boundary problems. The same was true for all varying intercepts distributions. Statistical significance for estimated regression parameters was assessed using *z*-tests, assuming standard normal distributed test-statistics under the null-hypothesis. Omnibus tests for entire interaction terms were performed using Wald tests for joint significance, assuming χ*^2^* distributed test statistics (see **Table 3**). In order to better understand the interaction effects, we computed and plotted predicted marginal means from the estimated model parameters for all interaction effects, corrected for multiple comparisons (Bonferroni; see **Figures 2**, **3**).

The relationship between attractiveness and distinctiveness as a function of age and emotion was explored using cross-random effects restricted cubic spline models. Restricted cubic splines allow for fitting flexible functions, without restricting them to a particular functional form, and thus were used to reveal potentially non-linear patterns in the relation between attractiveness and distinctiveness. In particular, we modeled attractiveness as a function of distinctiveness, using five knots located at the 5th, 27.5th, 50th, 72.5th, and 95th percentile of distinctiveness (Harrell, 2001). Varying the number and locations of knots did not yield substantial differences in the revealed patterns. The following model was fitted to sub-groups of interest:

$$y_{ijt}=\beta_{0}+\beta_{1}\psi_{ijt1}+\beta_{2}\psi_{ijt2}+\beta_{3}\psi_{ijt3}+\beta_{4}\psi_{ijt4}+\zeta_{i}+\zeta_{j}+\zeta_{it}+\varepsilon_{ijt}$$

where *y_ijt_* are the attractiveness ratings of the *i*th perceiver for the *j*th picture at time *t*, ψ*_ijt_*_1_ are the distinctiveness ratings, and ψ*_ijt2_*, ψ*_ijt3_*, and ψ*_ijt4_* are transforms of ψ*_ijt1_* according to the restricted cubic spline basis functions described in Harrell (2001).

All analyses were performed using the statistical analysis software Stata, version 13.1 (StataCorp, 2013). Throughout the manuscript, we present the actual *p*-values as long as they are larger than 0.001.

## Results

### Picture-Specific Data

**Table 1** summarizes raw means and SDs of facial attractiveness and distinctiveness ratings separately for age and gender of perceivers and faces. The practical aim of the present study was to provide researchers with picture-specific data for stimulus selection in future research. This picture specific data can be retrieved from http://faces.mpib-berlin.mpg.de (see also **Supplementary Material**). In particular, means and standard deviations of ratings for each face, organized by age and gender of perceiver and for each of the six emotions, for attractiveness and distinctiveness, respectively, are reported.

**Table 1:** Raw means (and SDs) of facial attractiveness and distinctiveness ratings separately for age and gender of perceivers and faces.

Perceivers		Faces M (SD)					
	
			Male			Female	
	
		Young	Middle-Aged	Older	Young	Middle-Aged	Older
		**Facial Attractiveness**					
Male							
	Young	30 (23)	22 (21)	19 (20)	45 (27)	25 (22)	18 (21)
	Middle-Aged	45 (21)	35 (20)	30 (21)	52 (24)	42 (23)	31 (21)
	Older	51 (23)	45 (23)	42 (24)	56 (23)	48 (25)	40 (24)
Female							
	Young	40 (27)	26 (21)	25 (22)	52 (26)	39 (24)	31 (23)
	Middle-Aged	40 (24)	32 (21)	27 (20)	47 (25)	37 (23)	31 (21)
	Older	44 (27)	33 (24)	25 (22)	48 (27)	39 (25)	28 (23)
		**Facial Distinctiveness**					
Male							
	Young	37 (23)	35 (23)	38 (24)	44 (24)	36 (23)	38 (25)
	Middle-Aged	46 (19)	42 (20)	43 (20)	48 (20)	45 (19)	45 (20)
	Older	46 (24)	43 (24)	42 (24)	49 (26)	45 (25)	41 (25)
Female							
	Young	42 (25)	35 (23)	37 (24)	48 (24)	42 (23)	43 (25)
	Middle-Aged	40 (23)	36 (23)	35 (24)	44 (22)	39 (22)	40 (23)
	Older	37 (25)	31 (23)	27 (22)	39 (25)	35 (24)	31 (23)


**Emotion-of-face–specific descriptive data is available from the authors upon request.**

### Error Components

**Table 2** shows results from cross-classified random effects models for attractiveness and distinctiveness, respectively. The error-components models (M1) show substantial variance for all variance components. Between-subject variance (standard deviation for perceivers) was substantially larger than the variance across rated faces for both attractiveness and distinctiveness. In addition, there was considerable within-subject variance across study sessions.

**Table 2:** Cross-classified random effects linear regression models.

	Facial Attractiveness		Facial Distinctiveness	
		
	M1	M2	M1	M2
**Age of Perceiver (Young)**				
Middle-Aged		5.271		-1.019
Older		9.614^∗∗∗^		-4.308
**Age of Face (Young)**				
Middle-Aged		-10.775^∗∗∗^		-0.521
Older		-16.995^∗∗∗^		1.597^∗^
**Gender of Perceiver (Male)**				
Female		-0.926		-3.497
**Gender of Face (Male)**				
Female		5.434^∗∗∗^		1.276^∗^
**Emotion of Face (Neutral)**				
Happy		5.987^∗∗∗^		5.681^∗∗∗^
Sad		-6.627^∗∗∗^		2.717^∗∗∗^
Angry		-9.357^∗∗∗^		4.956^∗∗∗^
Fearful		-9.697^∗∗∗^		9.381^∗∗∗^
Disgusted		-12.118^∗∗∗^		6.802^∗∗∗^
**Facial Attractiveness**				0.387^∗∗∗^
**Rating Count (Per Session)**		0.097^∗∗∗^		-0.005
**Rating Count (Per Session) Squared**		-0.000^∗∗∗^		0.000
**Constant**	36.726	39.863	40.155	23.587^∗∗∗^
*SD* Perceivers	15.040	14.449	14.709	11.240
*SD* Faces	9.347	5.496	5.065	3.464
*SD* Sessions	5.454	5.442	6.713	6.082
*SD* Residual Error	16.741	15.500	16.508	15.103
				
*N* Observations	134,942	134,942	134,942	134,942
*N* Perceivers	154	154	154	154
*N* Faces	171	171	171	171


**M1 = Error-components model; M2 = Model including predictor variables. ^∗^*p* < 0.05, ^∗∗^*p* < 0.01, ^∗∗∗^*p* < 0.001.**

In comparison with model M1, there was a substantial reduction in between-faces variance after inclusion of covariates (i.e., age, gender, emotion) in model 2 (M2) for attractiveness as indicated by the reduced standard deviation for faces in M2 (**Table 2**). This reduction was somewhat smaller for distinctiveness, reflecting the larger effects of the face-level covariates age, gender, and emotion of face for attractiveness than distinctiveness ratings. In comparison, the reduction in between-perceivers and sessions variance was smaller.

### Facial Attractiveness: Age, Gender, and Emotion Effects

#### Age

Partially supporting *Hypothesis 1a* regarding an age-of-perceiver main effect, older perceivers rated the faces as more attractive than young perceivers (fixed effects estimates in M2, **Table 2**). In addition, middle-aged perceivers gave higher attractiveness ratings than young perceivers, but this difference was not significant at the 5% level. The age-of-face main effect was significant for attractiveness. The fixed effects estimate from M2 in **Table 2** shows that young faces were rated as more attractive than middle-aged faces, and middle-aged faces as more attractive than older faces, supporting *Hypothesis 1b*. Furthermore, **Table 3** shows that the age of perceiver × age of face interaction was significant for attractiveness (χ^2^_(4)_ = 452.5; *p* < 0.001). That is, young perceivers, more than middle-aged and older perceivers, rated young faces as relatively more attractive than older and middle-aged faces (**Figure 2A**). This finding is in line with *Hypothesis 1c*.

**Table 3:** Wald tests for interaction terms.

Interaction Effect	χ*^2^*	*d.f.*	*p*
	**Facial Attractiveness**		
Age of Perceiver × Age of Face	452.5	4	<0.001
Age of Perceiver × Gender of Perceiver	11.6	2	0.003
Age of Face × Gender of Face	10.3	2	0.006
Age Perceiver × Emotion of Face	1067.0	10	<0.001
Age of Face × Emotion of Face	331.2	10	<0.001
	**Facial Distinctiveness**		
Age of Perceiver × Age of Face	548.3	4	<0.001
Age of Perceiver × Gender of Perceiver	2.37	2	0.307
Age of Face × Gender of Face	0.8	2	0.660
Age Perceiver × Emotion of Face	880.7	10	<0.001
Age of Face × Emotion of Face	163.6	10	<0.001


**Test results are based on omnibus tests for entire interaction terms; degrees of freedom correspond to the number of jointly performed tests.**

#### Gender

As shown in **Table 2**, the gender-of-perceiver main effect was not significant for attractiveness. The gender-of-face main effect estimate indicated that female faces were rated as more attractive than male faces. **Table 3** shows that the age of perceiver × gender of perceiver interaction was significant for attractiveness (χ^2^_(2)_ = 11.6; *p* = 0.003). In particular, young male perceivers gave the lowest attractiveness ratings, while older male perceivers gave the highest attractiveness ratings (**Figure 2C**). In contrast, there was no age-of-perceiver effect for women. Also, the age of face × gender of face interaction was significant for attractiveness (χ^2^_(2)_ = 10.3; *p* = 0.006). As shown in **Figure 2E**, while female young faces were rated as more attractive than male young faces, this gender-of-face effect was reduced in middle-aged and not present in older faces, supporting *Hypothesis 1d*.

#### Emotion

Substantial differences in ratings were observed across facial emotions, for attractiveness (**Table 2**). In particular, when compared to neutral faces, happy faces were rated as more attractive, while faces that displayed negative emotions were rated as less attractive, with disgusted faces rated as least attractive. This was in line with *Hypothesis 1e*. **Table 3** shows that for attractiveness the age of perceiver × emotion of face interaction was significant (χ^2^_(10)_ = 1067.0; *p* < 0.001). In particular, middle-aged and particularly older compared to young perceivers rated happy faces as relatively more attractive than all negative facial expressions (**Figure 3A**). **Table 3** also shows that the age of face × emotion of face interaction was significant for attractiveness (χ^2^_(10)_ = 331.2; *p* < 0.001): supporting *Hypothesis 1f*, higher attractiveness ratings for happy faces compared to all negative emotions were more pronounced in young than middle-aged or older faces (**Figure 3C**).

### Facial Distinctiveness: Age, Gender, and Emotion Effects

#### Age

The age-of-perceiver main effect for distinctiveness (controlling for variations in attractiveness) was not significant (**Table 2**). Older faces were perceived as more distinctive than young faces (**Table 2**). However, this effect was fairly small. The age of perceiver × age of face interaction was significant for distinctiveness (χ^2^_(4)_ = 548.3; *p* < 0.001). As illustrated in **Figure 2B**, older faces were perceived as more distinctive than young faces, however, only by young and middle-aged but not by older perceivers (supporting *Hypothesis 2b*).

#### Gender

As shown in **Table 2**, the gender-of-perceiver main effect was not significant for distinctiveness. The gender-of-face main effect was significant, though small, for distinctiveness: female faces were perceived as more distinctive than male faces. However, as shown in **Table 3**, neither the age of perceiver × gender of perceiver interaction (**Figure 2D**, χ^2^_(2)_ = 2.37; *n.s.*) nor the age of face × gender of face interaction was significant for distinctiveness (**Figure 2F**, χ^2^_(2)_ = 0.8; *n.s.*).

#### Emotion

Differences in ratings were observed across facial emotions for distinctiveness, but those were somewhat smaller than the differences in ratings across facial emotions seen for attractiveness (**Table 2**). Compared with neutral faces, fearful faces were rated as most distinctive, followed by disgusted, happy, angry, and sad faces. Thus, *Hypothesis 2c* was rejected. The age of perceiver × emotion of face interaction was significant for distinctiveness (χ^2^_(10)_ = 880.7; *p* < 0.001). As shown in **Figure 3B**, the age of perceiver groups largely agreed on distinctiveness ratings for neutral and happy emotions. However, young, and to some extent middle-aged, perceivers rated faces displaying negative expressions, and particularly fear and disgust as more distinctive than the other negative facial expressions. The age of face × emotion of face interaction was also significant but the effects were small (χ^2^_(10)_ = 163.6; *p* < 0.001; **Figure 3D**), pointing toward increased ratings of distinctiveness for fearful and disgusted older compared to middle-aged and young faces.

### Relations Between Attractiveness and Distinctiveness

**FIGURE 4:**
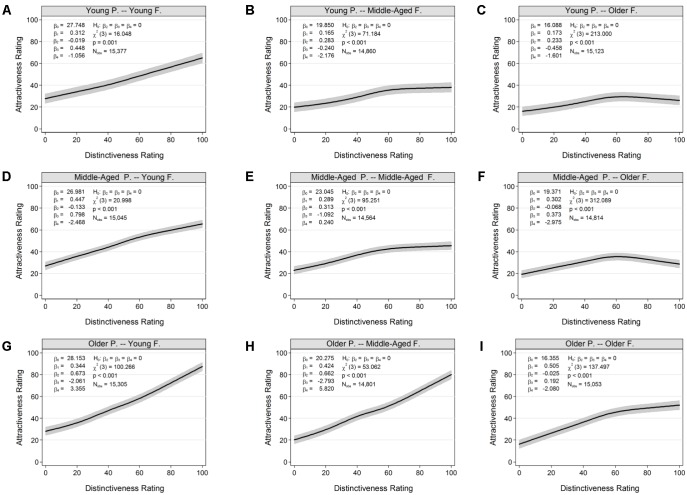
Variations in the relation between attractiveness and distinctiveness ratings by age of perceiver and face: **(A)** Young Participants, Young Faces. **(B)** Young Participants, Middle-Aged Faces. **(C)** Young Participants, Older Faces. **(D)** Middle-Aged Participants, Young Faces. **(E)** Middle-Aged Participants, Middle-Aged Faces. **(F)** Middle-Aged Participants, Older Faces. **(G)** Older Participants, Young Faces. **(H)** Older Participants, Middle-Aged Faces. **(I)** Older Participants, Older Faces. Black lines show expected values of attractiveness, conditional on distinctiveness. Shaded areas indicate 95% confidence regions. Results were obtained from separate fits of multilevel restricted cubic spline models (Eq. 1). P = Perceiver; F = Faces.

**FIGURE 5:**
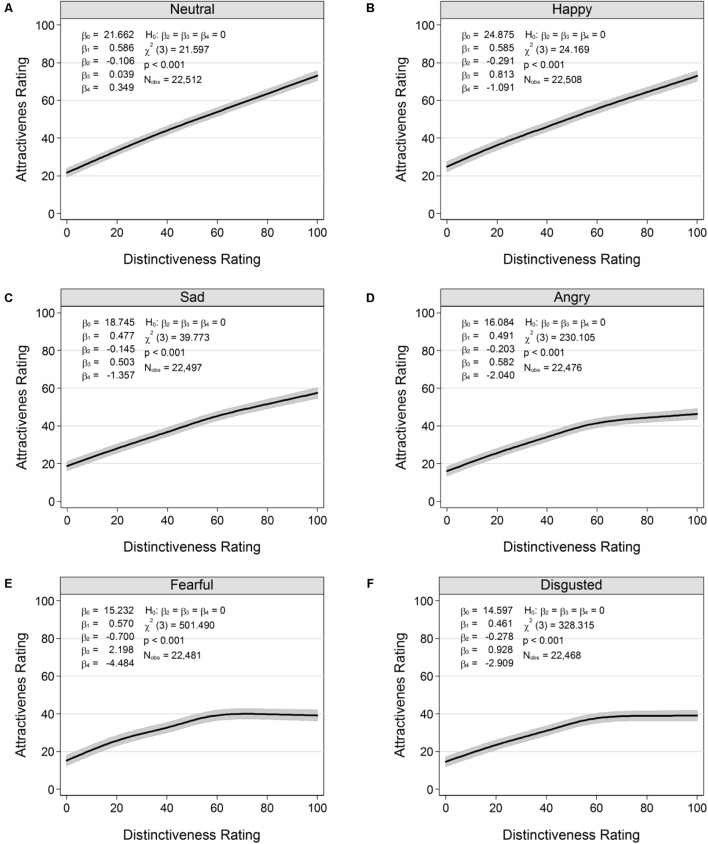
Variations in the relation between attractiveness and distinctiveness ratings by emotion of face: **(A)** Neutral. **(B)** Happy. **(C)** Sad. **(D)** Angry. **(E)** Fearful. **(F)** Disgusted. Black lines show expected values of attractiveness, conditional on distinctiveness. Shaded areas indicate 95% confidence regions. Results were obtained from separate fits of multilevel restricted cubic spline models (Eq. 1). P = Perceiver; F = Faces.

An exploratory aim in the present study was to advance the current knowledge on the relationship between attractiveness and distinctiveness and how this relationship may vary by age and emotion. Results of this analysis suggested linear and non-linear relationships between attractiveness and distinctiveness, conditional on age and emotion. In particular, distinctiveness of young faces monotonically increased with increasing levels of attractiveness in young (**Figure 4A**), middle-aged (**Figure 4D**), and particularly older (**Figure 4G**) perceivers. For middle-aged (**Figures 4B,E,H**) and older (**Figures 4C,F,I**) faces, there was a similar positive relationship (i.e., increasing levels of distinctiveness were associated with increasing levels of attractiveness) at low levels of distinctiveness. However, with increasing levels of distinctiveness, there was a “flattening out” of attractiveness ratings for middle-aged faces, and decreasing levels of attractiveness for older faces, particularly in young and middle-aged perceivers. For neutral (**Figure 5A**), happy (**Figure 5B**), and sad (**Figure 5C**) faces there was a monotonically increasing relationship between attractiveness and distinctiveness. In contrast, for angry (**Figure 5D**), fearful (**Figure 5E**), and disgusted (**Figure 5F**) faces there was a “flattening out” of attractiveness ratings, with increasing levels of distinctiveness.

## Discussion

This study considered effects of age and gender of perceiver and effects of age, gender, and emotion of face on subjective perception of facial attractiveness and distinctiveness. The central findings of the study support the notion that perceived attractiveness and distinctiveness constitute developmentally relevant constructs. In particular, (1) age of perceiver affected attractiveness ratings; (2) attractiveness and distinctiveness varied by age of face in interaction with age of perceiver; (3) age affected attractiveness more negatively for female than male faces; (4) facial emotion affected attractiveness and distinctiveness; and (5) attractiveness and distinctiveness were positively correlated with some variation in this relationship by age and emotion.

Below, we discuss this study’s novel findings in relation to our original hypotheses and with respect to classic and recent work pertaining to prototyping processes, social categorization, neurobiological factors, age differences in goal-directed perception, social motivation, and in-group bias, for a developmental interpretation of our findings. We conclude with reflections on theoretical and practical implications as well as study limitations and future directions.

### Age of Perceiver Influenced Attractiveness Ratings

As expected, overall older compared to young perceivers rated faces as more attractive. This finding can be interpreted in the context of the *expertise hypothesis* (Goldstein and Chance, 1980; Bartlett and Leslie, 1986), according to which individuals with greater face expertise perceive attractiveness in all faces as a reflection of the appreciation for beauty of faces more generally. In contrast, individuals with less expertise have a narrower view of what makes a face attractive. Given their extended lifespan, older adults have gained greater expertise with faces compared to young adults, and hence may have developed a broader concept of facial attractiveness. This may underlie their greater tendency to endorse attractiveness for faces as observed in our study.

Facial attractiveness perception appears to a large extent to be a result of species-type psychological adaptations, which evolved because of preference for healthy and fertile mates (Thornhill and Gangestad, 1999). However, given the cross-sectional design of our study it is possible that the greater attractiveness ratings given by older than young perceivers (partly) reflected a cohort difference (*cohort hypothesis*; Foos and Clark, 2011). For example, there is evidence that faces that were regarded as attractive in the early decades of the 20th century are quite different from those regarded as attractive today. That is, individuals of different ages may judge faces differently because they value and hold different standards for different features and facial configurations, influenced by historical beauty standards that are specific to the times in which they have lived (DeSantis and Sierra, 2000). Future cross-sequential designs are necessary to dissociate age and cohort effects in facial attractiveness ratings (Schaie, 1994).

Relatedly, cultural similarities and differences need to be considered in future research. This will allow disentangling effects that are universal (and hence presumably biologically based) from effects that are shaped through socio-cultural influences. Nowadays, and maybe particularly in modern western cultures, youth is highly valued. This culminates in high rates of surgical alterations and application of cosmetics in Western cultures (Bruce and Young, 1998). In contrast, in some other (Eastern) cultures, wisdom and experience, which are more associated with advanced age than youth, are highly valued, which may reduce the desire to appear young in these cultures.

### Age of Face and Perceiver Influenced Attractiveness and Distinctiveness Ratings

As expected, young faces received higher attractiveness ratings than middle-aged and older faces. Although older and middle-aged compared to young perceivers generally rated faces of all ages as more attractive, young perceivers gave relatively higher attractiveness ratings to young compared to middle-aged and older faces. This differential effect is in line with the expertise hypothesis mentioned above assuming that perceivers from various age groups, due to differences in experience, may hold partly different concepts of attractiveness. This interaction effect could additionally reflect a self-serving bias in young perceivers toward their own age group (Wernick and Manaster, 1984; McLellan and McKelvie, 1993; Ebner, 2008), which would be in line with the s*imilarity hypothesis* (Sappenfield and Balogh, 1970), according to which individuals judge those that are perceived as being more similar to themselves as more attractive to promote positive self-views.

However, it could also be that the more negative evaluation of older relative to young faces by young perceivers is due to young perceiver’s greater visual experience with young compared to older faces. That is, older faces may deviate more from young perceiver’s facial prototypes and may in turn trigger an anti-age bias. Rhodes et al. (2003) suggested that experience of faces shapes what is considered prototypical, which in turn influences what is perceived as being attractive or distinctive. However, this would not explain why the effect was only present in young but not older perceivers, given that evidence suggests that both young and older adults report more frequency of exposure with their respective age group (Ebner and Johnson, 2009).

Rather, motivational factors may have influenced perception of attractiveness in our study (i.e., goal-directed perception). One of the primary social motivations in young adulthood is to develop romantic relationships (Zimmer-Gembeck, 2002). Thus, face attractiveness may constitute a particularly relevant facial feature for young adults. In contrast, face attractiveness may play less of a role in older adults’ processing of faces, as mate selection becomes less relevant in older age (Fredrickson and Carstensen, 1990; Lindau et al., 2007). Thus, facial attractiveness may constitute a highly salient dimension in young faces for young perceivers but less so for older faces and in older perceivers (Lin et al., 2016, 2017). This interpretation is in line with the *interest hypothesis* (Rodin, 1987) that states that one pays closer attention to those who seem of interest in pursuit of one’s personal goals and agenda, while disregarding those of less interest and relevance.

We also observed effects of age of face and interactions with age of perceiver for distinctiveness ratings. In particular, older faces were perceived as more distinctive than young faces by young and middle-aged but not by older perceivers. This may have been due to increased wrinkles and other age-related facial change. This interesting effect was small, however, and needs to be replicated in the future.

Taken together, our findings suggest that age-specific prototyping effects and age-based in-group biases are at work during the evaluation of facial features, at least in young adults. In addition, age differences in goals may modulate perception of attractiveness in that they affect what individuals consider in their evaluations.

### Age Influenced Attractiveness More Negatively for Female Than Male Faces

As hypothesized, age affected attractiveness ratings more pronouncedly and negatively for female than male faces. This finding can be interpreted in the context of the *crone hypothesis* according to which older women generally are perceived more negatively than older men (Healey, 1993; Hurd, 1999). While gray hair in men is often viewed as reflecting higher social standing; in women it is perceived as a sign of loss of fertility associated with old age. Portrayals of older women in the media are typically less positive than those of older men (Dail, 1988; Ginn and Arber, 1991). Our study suggests that these negative connotations may extend to perception of facial attractiveness (Foos and Clark, 2011).

### Facial Emotion, in Interaction With Age of Face and Perceiver, Influenced Attractiveness and Distinctiveness Ratings

Considering facial emotion, we observed substantial differences in attractiveness ratings in line with our hypothesis. In particular, happy faces were rated as most attractive, while disgusted faces were rated as least attractive. This finding supplements evidence by O’Doherty et al. (2003) that faces displaying positive expressions were perceived as more attractive and were associated with the reward network. High attractiveness ratings for happy faces were particularly pronounced in middle-aged and older perceivers and in young and female faces.

Also, there were significant differences in distinctiveness ratings across facial emotions. Compared with neutral faces, fearful faces were rated as most distinctive, followed by disgusted, happy, angry, and sad faces. This was counter our hypothesis that happy faces may be perceived as most distinctive. Happy faces in our stimulus material were the only expressions that showed teeth. A previous study had found that distinctiveness of such local features can promote pop-out effects (Calvo and Marrero, 2009). However, our study varied methodologically from this previous work in that it used an age- and gender-heterogeneous set of naturalistic facial emotion expressions and presented one face at a time in the context of an explicit rating task, as opposed to the multiple-face arrays as used by Calvo and Marrero (2009). Also, while perceivers largely agreed on distinctiveness ratings for neutral and happy emotions, older and middle-aged compared to young perceivers rated faces displaying negative emotions (particularly fear and disgust) as more distinctive.

The present study did not explicitly test prototype theory and social categorization. However, both accounts offer interesting perspectives on the effects we observed. Prototype theory asserts that a basic feature of human cognition is the creation of category prototypes to increase utility and efficiency of information processing (Rosch, 1987). There has been evidence that category prototypes are cognitively pleasing (Halberstadt and Rhodes, 2000). That is, good exemplars (prototypes) of a specific group make a face easier to process and more attractive (Winkielman et al., 2006). According to the prototype account, face categories such as age, gender, and race are evaluated separately and thus different explanations may apply for perception of attractiveness and distinctiveness for these different types of faces.

Relatedly, there is a debate in the current literature as to the extent to which facial features are processed independently vs. interdependently. Some models of face perception emphasize independent processing of functionally different aspects of faces (e.g., age, race, identity, gender, emotion; Bruce and Young, 1986; see also Le Gal and Bruce, 2002). In contrast, other models propose interdependent processing of facial features, supported by neuroimaging evidence (Haxby et al., 2000). In particular, physical aspects of different face patterns are coded by the inferior occipital gyri (Rotshtein et al., 2004). This takes place before the face processing system bifurcates into two relatively specialized, but interacting, systems: the lateral fusiform gyrus for processing invariant face cues such as identity gender, or race and the superior temporal sulcus for processing changeable facial cues such as gaze and head direction, or expression (cf. Calder and Young, 2005; Ganel et al., 2005). Further supporting independent processing is research demonstrating faster gender classification for familiar compared to unfamiliar faces (Rossion, 2002) and gender- and race-contingent expression aftereffects (Bestelmeyer et al., 2010). That is, visual adaptation to angry faces of one gender and fearful faces of the other gender simultaneously caused faces of the first gender to appear less angry and faces of the other gender to appear less fearful post-compared to pre-adaption (Hsu and Young, 2004; Webster et al., 2004; Bestelmeyer et al., 2010, for similar findings). It is likely that equivalent effects apply to age of face. Future studies are needed to systematically determine the extent to which young, middle-aged, and older perceivers’ ratings of attractiveness and distinctiveness are made in relation to same-gender and same-age rather than overall face prototypes (Baudouin and Gallay, 2006).

Similarly, social-cognitive research supports a role of social categories in face processing. Activation of a social category can bias perception and memory for faces in a category-consistent direction (Young et al., 2009). For example, social categories elicit perceptual assimilation to prototypes, which makes a face more similar to faces of other members of the same category (Valentine, 2001; Corneille et al., 2007). Thus, the presentation of face stimuli of distinctively different ages and faces of different gender may have instantiated an inter-group bias and influenced evaluations of facial attractiveness and distinctiveness. This induction of category-based assimilation via mixed stimuli presentation and thus creation of an intergroup context (age- and gender-based) with effects on perceived attractiveness and distinctiveness will have to be specifically targeted in future research.

### Age and Emotion Affected Relationship Between Attractiveness and Distinctiveness

To our knowledge, the present study is the first to examine the attractiveness-distinctiveness link from a developmental perspective and with respect to facial emotion variations. Research on distinctiveness and its relationship to attractiveness is particularly relevant in the context of determining influences of these facial features on face memory. We were able to contribute to the current knowledge by showing that the relationship between attractiveness and distinctiveness varied as a function of age and emotion. In particular, these two constructs were positively and linearly associated for young, middle-aged, and particularly older perceivers, and for neutral, happy, and sad faces. However, at increasing levels of distinctiveness we found a “flattening out” of attractiveness ratings for angry, fearful, and disgusted faces and middle-aged faces, and decreasing levels of attractiveness for older faces, particularly in young and middle-aged perceivers.

### Theoretical and Practical Implications

Our study extends previous knowledge and has several practical implications. In particular, our results qualify current social-cognitive and evolutionary approaches that do not systematically consider age, gender, and emotion variations in the perception of facial features. By adopting a developmental perspective, our study contributes to the still limited knowledge of age-related differences in goal-directed perception, social goals, and in-group biases that affect face perception. Knowledge gained from our results could be practically applicable in the context of impression formation and person memory in everyday social interactions and in eye witness testimony, mating behavior, job employment, and career opportunities, or with regard to determination of efficacy of advertising strategies and in looks improvement via plastic surgery (Bashour, 2006).

The faces database we had available for this research took an adult lifespan approach, also including (typically understudied) middle-aged faces and middle-aged perceivers. Thus, the present study generates new knowledge about how effects vary more gradually from young, over middle-aged, to older adulthood. These findings have potential to advance theory in adult developmental research on face perception (with regard to facial attractiveness and distinctiveness).

Compared with previous research (Alicke et al., 1986; Furnham et al., 2001; Ebner, 2008; Foos and Clark, 2011; Kwart et al., 2012), our study used a larger sample, a larger set of facial stimuli, a more extensive response range, and a modeling statistical approach which allowed accommodation for the cross-classified data structure. We systematically varied facial emotion expression in addition to examining face age and gender in their effects on perceived attractiveness and distinctiveness and our study design allowed dissociation of variations of attractiveness from those of distinctiveness.

Importantly, with this publication, we make available raw means and standard deviations of attractiveness and distinctiveness ratings for each face and separately for age and gender of perceivers. This picture-specific data will allow researchers interested in a broad range of developmental questions pertaining to processing of faces and emotions the selection of adequate experimental stimuli from the FACES Lifespan Database (Ebner et al., 2010) based on normative information. Information about the FACES database and registration formalities for download of the face images and accompanying normative data can be obtained from http://faces.mpib-berlin.mpg.de.

### Limitations and Future Directions

One purpose of the present study was to examine perceiver and face characteristics on *subjective perception* of facial attractiveness and distinctiveness. Age, gender, and emotion effects on *objective measures* of distinctiveness such as related to symmetry of facial composition (Rhodes et al., 1999; Komori et al., 2009) or deviations of facial proportions from average calculated using landmark analysis (Bookstein, 1991) are currently understudied. Distinctiveness can be defined as the proximity of faces to other faces in a multidimensional face space (Valentine, 2001). However, this conceptualization of distinctiveness is different from the distance from the norm, or average face (i.e., averageness) and from the subjective measures applied in the present context. It will be exciting in future research to determine the extent to which age, gender, and emotion affect objective measures of distinctiveness (e.g., symmetry, averageness/prototype). It will also be crucial to describe relations between subjective and objective facial features to better understand a possible “halo” or bleed-through effect from facial attractiveness to distinctiveness and person characteristics such as trustworthiness or personality (Dion et al., 1972; Eagly et al., 1991; Griffin and Langlois, 2006).

Our study exclusively used static photographs of facial expressions. Although faces are important contributors to overall physical attractiveness (Alicke et al., 1986; Furnham et al., 2001), bodily displays in combination with facial displays contribute to perception of attractiveness, but are currently largely ignored in the literature (Mueser et al., 1984; Peters et al., 2007). Attractiveness ratings of static, posed faces may not be analogous to the more ecologically valid dynamic face stimuli that are frequently used in other areas of attractiveness research such as in the context of real-life or laboratory-based social interactions. Rubenstein (2005) found a low correspondence in attractiveness ratings of the same face across dynamic vs. static face presentation and characteristics such as emotional expression, age, and facial adiposity may be more salient in dynamic than static presentations as future research will be able to show (Holland et al., 2018). Current literature on facial emotion expression also use additional positive and negative emotions than the ones examined in the present study (Bänziger et al., 2012).

We did not collect explicit information about reproductive status or measure hormone concentrations. Presumably, based on the participants’ chronological age, young women in our study were at peak fertility, middle-aged women were peri-menopausal, and older women were post-reproductive. These differences in reproductive status may underlie some of the differences seen across age and gender in our data. This interpretation is in line with suggestions from hormone theory of facial attractiveness that perception of attractiveness vary with displayed hormone markers and the reproductive and hormonal state of the perceiver (Johnston et al., 2001; Saxton et al., 2006). Supporting this idea is evidence that female preference for male facial characteristics coincides with the menstrual cycle. In particular, women in the follicular compared to luteal phase of their menstrual cycle were more attentive to phenotypic markers indicating immune-competence and prefer stereotypically “masculine” faces characterized by extreme testosterone markers (Penton-Voak et al., 1999; see also Gangestad and Thornhill, 1998; Havlicek et al., 2005; Feinberg et al., 2006). Puts et al. (2013) provided direct evidence that estradiol and progesterone levels were implicated in greater perceived attractiveness of women during their peak fertility by both male and female raters. Saxton et al. (2006) furthermore showed that young female adults and adolescents judged male facial and vocal attractiveness quite concordantly, while this relationship was not manifested in pre-menarchal children. These findings suggest that attractiveness judgments vary as a function of the individual’s life stage (Saxton et al., 2009).

Research on competitor derogation and its effects on perceived facial attractiveness is also relevant in this context. Competitor derogation refers to any act performed to decrease, relative to oneself, the value (e.g., appearance, personality) of a rival as a potential mate (Buss and Dedden, 1990). It has been suggested to constitute a reflection of indirect aggression and intra-sexual competition (Fisher et al., 2009). In particular, negative statements made by a female, particularly an attractive female, about a female competitor can reduce male’s evaluations of that competitor’s facial attractiveness. There is evidence that competitor derogation in women is particularly pronounced during peak fertility (Fisher, 2004). This is in line with research showing women’s ratings of female faces decreased during ovulation while post-menopausal women expressed stronger preferences for feminine female faces (Jones et al., 2005).

This evidence combined suggests that reproductive status and hormone concentration can impact perception of facial features. However, a recent very large longitudinal study came to no concluding evidence that women’s hormonal status predicts their preferences for masculinity in faces (Jones et al., unpublished). We believe that future studies will benefit from collecting information about menstrual cycles and conducting hormone assays and collecting information about sexual orientation to systematically examine influences of these variables on face perception in age- and sex-heterogeneous samples.

Attractiveness ratings were always given first for each image, thus it is unlikely that these ratings were influenced by ratings on the other dimensions. Further, in our analysis of the distinctiveness data, we statistically controlled for variations in attractiveness ratings. However, our study design cannot fully exclude the possibility that rating of one dimension may have affected rating of another dimension within and between face identities, as each face was rated on more than just one dimension in the current study’s within-group design. Future research is warranted to address possible spill-over effects.

## Conclusion

This study presents data on facial attractiveness and distinctiveness that is crucial for researchers for facial stimulus selection. We hope that making this data freely available to the research community will further spur use of the FACES Lifespan Database for scientific purposes in well-controlled experiments. The pattern of results reported in this study supports the idea that facial attractiveness and distinctiveness constitute developmentally relevant constructs and that the social and biological relevance of these facial features may change in line with developmental tasks across the lifespan. Our findings suggest age differences in social goals, prototyping, social categorization, and in-group biases at work during face perception. Moving forward, it appears particularly fruitful to apply the present findings to the context of face memory and with regard to affective behavior during social interactions in naturalistic lab-settings or real-life contexts (e.g., in legal domains, in advertising).

## Author Contributions

NE and UL designed the study and supervised the data collection. JL analyzed the data and reported the findings. NE, MV, MR, and TL wrote the manuscript.

## Conflict of Interest Statement

The authors declare that the research was conducted in the absence of any commercial or financial relationships that could be construed as a potential conflict of interest.

## Supplementary Material

DATA SHEET 1 Perceived facial attractiveness.

DATA SHEET 2 Perceived facial distinctiveness.

DATA SHEET 3 Read me file for perceived facial attractiveness and perceived facial distinctiveness.
