# Supplementary material for: An Adult Developmental Approach to Perceived Facial Attractiveness and Distinctiveness
Source: Front Psychol. 2018 May 7;9:561. doi: 10.3389/fpsyg.2018.00561 (PMC5949528; doi:10.3389/fpsyg.2018.00561)
Supplement: DATA SHEET 3 — Read me file for perceived facial attractiveness and perceived facial distinctiveness. [file Data_Sheet_3.DOCX]

-----------------------

Legend for .xlsx files:

-----------------------

Mean_YM --> Mean ratings for young male perceivers

Mean_YF --> Mean ratings for young female perceivers

Mean_MM --> Mean ratings for middle-aged male perceivers

Mean_MF --> Mean ratings for middle-aged female perceivers

Mean_OM --> Mean ratings for older male perceivers

Mean_OF --> Mean ratings for older female perceivers

SD_YM --> Standard deviation of mean for young male perceivers

SD_YF --> Standard deviation of mean for young female perceivers

SD_MM --> Standard deviation of mean for middle-aged male perceivers

SD_MF --> Standard deviation of mean for middle-aged female perceivers

SD_OM --> Standard deviation of mean for older male perceivers

SD_OF --> Standard deviation of mean for older female perceivers

To allow for easy reference, entries are listed according to their identifying labels in the FACES database.

-------------------------------------------------------------------------------------------------------------------------
